# Bone metastasis detection method based on improving golden jackal optimization using whale optimization algorithm

**DOI:** 10.1038/s41598-023-41733-x

**Published:** 2023-09-12

**Authors:** Omnia Magdy, Mohamed Abd Elaziz, Ahmed Elgarayhi, Ahmed A. Ewees, Mohammed Sallah

**Affiliations:** 1 Applied Mathematical Physics Research Group, Physics Department, Faculty of Science, Mansoura University, Mansoura, 35516 Egypt; 2 Department of Mathematics, Faculty of Science, Zagazig University, Zagazig, 44519 Egypt; 3 Faculty of Computer Science and Engineering, Galala University, Suez, 435611 Egypt; 4 Artificial Intelligence Research Center (AIRC), Ajman University, Ajman, UAE; 5 Department of Electrical and Computer Engineering, Lebanese American University, Byblos, Lebanon; 6 MEU Research Unit, Middle East University, Amman, Jordan; 7 Department of Computer, Damietta University, Damietta, 34517 Egypt; 8 Department of Physics, College of Sciences, University of Bisha, P.O. Box 344, Bisha , 61922 Saudi Arabia

**Keywords:** Image processing, Machine learning

## Abstract

This paper presents a machine learning-based technique for interpreting bone scintigraphy images, focusing on feature extraction and introducing a new feature selection method called GJOW. GJOW enhances the effectiveness of the golden jackal optimization (GJO) algorithm by integrating operators from the whale optimization algorithm (WOA). The technique’s performance is evaluated through extensive experiments using 18 benchmark datasets and 581 bone scan images obtained from a gamma camera, including 362 abnormal and 219 normal cases. The results highlight the superior predictive effectiveness of the GJOW algorithm in bone metastasis detection, achieving an accuracy of 71.79% and specificity of 91.14%. The contributions of this study include the introduction of a new machine learning-based approach for detecting bone metastasis using gamma camera scans, leading to improved accuracy in identifying bone metastases. The findings have practical implications for early detection and intervention, potentially improving patient outcomes.

## Introduction

Bone metastasis is a common cancer consequence caused by the propagation of cells from particular primary malignancies. Specifically, cancer spreads from the lung, breast, and prostate to the bone^1^. Treatment choices are significantly impacted by early diagnosis of bone metastases. While also being a critical component in disease progression and quality of life. Bone metastases are typically diagnosed using a combination of symptoms and signs (fractures, bone pain, lack of appetite, disorientation, fatigue, nerve difficulties, hypercalcemia, etc.) and imaging data^2^. Various imaging techniques, such as magnetic resonance imaging (MRI), positron emission tomography (PET), computed tomography (CT), and X-rays, are currently utilized in clinical practice to diagnose bone metastases, but bone scanning by gamma camera is regarded as the gold standard^3^.

Artificial intelligence (AI) has already demonstrated significant potential in tackling this issue by means of automated diagnosis using medical imaging. The development of artificial neural networks (ANNs) resulted in an explosion of statistical model-based machine learning (ML)^4^, the machine learning application to calculate the size and position of the lesion area can significantly reduce doctor workload, reduce misdiagnosis and missed diagnoses, and increase diagnosis accuracy, depending on the effectiveness of each model. For instance, Salman et al.^5^ offered an ANN method that was interbred with three metaheuristic techniques, namely Fireworks Algorithm (FWA), particle swam optimization (PSO), and genetic algorithm (GA) which are created by locating the ideal set of ANN weights to enhance accuracy performance. They used 569 Wisconsin Breast Cancer datasets, 768 Pima Indian Diabetes datasets, 345 Liver Disorder datasets, 195 Parkinson’s datasets, 306 Haberman Surgery Surviva datasets, and 801 Gene expression cancer datasets.

These studies still have significant flaws, like poor accuracy, this paper proposes adapting two algorithms to raise the efficiency of our model, and evaluated it against other algorithms, showing that the new algorithm has better results in accuracy, sensitivity, and specificity than other algorithms, and shows the potential of raising the efficiency of the model in increase diagnosis accuracy. This led us to suggest a different approach for detecting bone metastases that rely on optimizing the performance of the golden jackal algorithm (GJO) with the help of the Whale optimization algorithm (WOA).

The proposed model starts by extracting the features from the images of Bone metastasis using handcrafted features. Followed by determining the relevant features from those extracted ones. This process is performed through a set of steps. The first step is to produce a set of the population and then assess the efficiency of these solutions using the fitness that depends on the error of classification. Then using the modified version of GJO based on the operators of WOA that are used to enhance the exploitation ability of GJO. After that, the best solution is returned from the updating process was to reduce the size of a testing set and evaluate the quality of this reduction using performance measures.

This study’s primary contributions can be summed up as below:

1. Suggest gamma camera scanned bone metastasis detection method using a machine learning method.
2. Improve the performance of Golden jackal optimization (GJO) based on the operators of the Whale optimization algorithm (WOA).
3. Assess the efficiency of the developed feature selection method using real-world Bone metastasis datasets.

The remaining part of the paper is structured as follows. Bone metastasis detection method and metaheuristic (MH) techniques are given in Sects. “The literature of ML for bone metastasis detection” and “The literature of application of MH techniques”. A quick summary of WOA and GJO is given in Sects. “The whale optimization algorithm” and “Golden jackal optimization”. The proposed technique and thorough exegesis of the suggested GJOW are provided in Sect. “Suggested feature selection algorithm”. The experimental results from different test datasets are reported in Sect. “Results”. Then, conclusions are reported in Sect. “Conclusion and future work”.

## Related work

In this section, we present the related works for the bone metastasis detection and metaheuristic.

### The literature of ML for bone metastasis detection

There are some AI techniques that have been developed to handle bone metastasis detection. For example, Sharma et al.^6^ carried out two trials applied to two machine learning models, one with a histogram of oriented gradients feature set and another without hog features, the study’s dataset contains 105 images. Eid and Sauber^7^ proposed an algorithm that relies on integral using two methods to improve the skeletal scintigraphy image employing 465 datasets, namely, the Salp Swarm algorithm (SSA) and the neutrosophic set (NS). Liu et al.^8^ developed a machine learning-based random forest (RF) algorithm model. The accuracy score, specificity, recall rate, and area under the receiver operating characteristic curve (AUC) were used to assess and contrast the effectiveness of the RF paradigm and the other paradigm in terms of making predictions by using 17,138 patients.

Nakajima et al.^9^ employed software for calculating BSI, and compared to the original database, The multi-institutional database significantly improved bone metastases detection, highlighting the significance of having a sufficient number of training databases with a variety of cancer systems, a database including 1532 patients. Avula et al.^10^ utilized the k-means clustering algorithm to divide the bone images. In order to detect bone cancer, the segmented picture is further remedied by calculating the mean intensity of the discovered area. Ranjitha et al.^11^ developed a method for identifying bone malignant growths in ultrasound scans of bones using a KNN classifier and k-means segmentation to identify a bone illness. Sinthia et al.^12^ developed a technique for recognizing bone tumors using MRI. The average filter and the bilateral filter are two pre-processing techniques that are included in the suggested method to eliminate noise and smooth pictures. By computing the k-means approach to calculate the mean intensity and malignancy size, the existence of bone cancer is checked on the MRI bone cancer images to identify its stage.

Satheesh et al.^13^ advocated using a dataset from a clinical dataset to detect bone cancer. Here, the suggested design consists of two steps for more accurate disorder prediction. The Gray-Level Co-occurrence Matrix (GLCM) method is used in the first step to extract features from a segmented bone image that are based on statistical texture. The second stage involves classifying the extracted features using KNN and a decision tree algorithm. Zhang^14^ proposed a novel PSO-based unsupervised feature selection method, called filter-based bare-bone particle swarm optimization algorithm (FBPSO). To hasten the algorithm’s convergence, two filter-based techniques are suggested. A local filter search method based on feature redundancy is utilized to increase the swarm’s capacity for exploitation, while a space reduction strategy based on average mutual information is employed to quickly remove irrelevant and tangentially relevant features. Bhukya et al.^15^ proposed a system for automatically detecting bone cancer to help physicians find the disease early and get patients the treatment they need. A fuzzy C-Means (FCM) and M3-filtered segmentation method on the basis of support vector machines (SVM) is proposed for the detection of bone malignancies.

In addition, Shukla et al.^16^ used image segmentation methods such as Prewitt, Sobel, k-means, Canny, and Region Growing, it is stimulable for the interpretation of X-ray and MRI images. The results of edge-based and region-based image segmentation methods applied to X-ray images to identify osteosarcoma cancer on bone are also presented.

### The literature of application of MH techniques

Recently, the MH techniques had more attention since they have been applied to different applications. For example, H. Mohammadzadeh et al.^17^ proposed a new approach based on the concept of agents and MASs, It is referred to as Metaheuristic (MAMH) method. Several primary and robust metaheuristic algorithms are viewed as detached agents in the proposed method, each attempting to achieve its own goals while competing and working with others to achieve common goals. Altogether, the suggested method was examined on 32 complicated benchmark functions, and the results demonstrated its usefulness and power in tackling high-dimensional optimization issues.

Gharehchopogh et al.^18^ examined the sparrow search algorithm (SSA), one of the modern and reliable algorithms for resolving optimization issues. And covered all of the SSA literature on improvement, variants, optimization, and hybridization. Piri, J. et al.^19^ originally used a technique called artificial gorilla troop optimization (DAGTO) to handle FS jobs in the healthcare industry. Depending on how many and what kind of objective functions there are, and implemented four variations of the suggested method, including: (1) single-objective (SO-DAGTO), (2) bi-objective (filter wrapper hybrid) (MO-DAGTO2) (3) bi-objective (wrapper) (MO-DAGTO1) and (4) tri-objective (filter wrapper hybrid) (MO-DAGTO3) for identifying pertinent features in diagnosing a specific disease. To increase population diversity and hasten convergence, they offer a superb gorilla initialization technique based on label mutual information (MI). Ten medical datasets are considered to verify the performance of the offered approaches.

Shishavan S.T. and F.S. Gharehchopogh^20^ enhanced Cuckoo Search Optimization (CSO) technique with a Genetic technique (GA) for community discovery in complex networks. GA has been highly successful in increasing exploration and exploitation by detecting communities in complex networks. Premature convergence, delayed convergence, and becoming stuck in the local trap are all issues with the CSO algorithm. GA operators were utilized dynamically to improve the speed and accuracy of the CSO. The proposed algorithm was tested with Artificial Bee Colony (ABC), GA, CSO, and grey wolf optimizer (GWO), with various iterations in the modularity and NMI criterion. The results reveal that in most comparisons, the suggested algorithm outperformed the fundamental comparative algorithms. Gharehchopogh, F.S.^21^ presented a review of different usages of QC in metaheuristics This review also includes a taxonomy of Quantum-inspired metaheuristic algorithms in optimization issues, as well as discussions of their applications in engineering and science. Their main objectives are to provide an overview of and review the applications of quantum-inspired metaheuristic algorithms.

Shen Y. et al.^22^ presented a novel population evolution technique to assist MEWOA in improving its global optimization abilities and avoiding local optimumMEWOA is compared to five modern WOA variations and seven fundamental metaheuristic algorithms over 30 benchmark functions with, respectively, dimensions of 100, 500, 1000 and 2000. On the majority of benchmark functions, it has been found that MEWOA exhibits shorter runtime, achieves faster convergence speed, and offers higher solution accuracy than other methods. Ayar, M. et al.^23^ introduced a novel chaotic-based divide-and-conquer (CDC) algorithm to pick the best features out of a feature collection (the UCI Arrhythmia Dataset). In terms of accuracy, sensitivity, specificity, and F-measure, the proposed method produced performance rates of 88.21%, 89.41%, 87.64%, and 86.54%, respectively.

Gharehchopogh F.S. et al. ^24^examined a new meta-heuristic algorithm known as the Slime Mould algorithm (SMA) from many optimization points. The fluctuating behavior of slime mold in nature led to the creation of the SMA algorithm. In the aforementioned regions, SMA is used at rates of 15, 36, 7, and 42%, respectively. The results support the assertion that SMA has been successfully used for numerous optimization issues As a result, it is hoped that academic scientists, professionals, and engineers will find this study useful. Mohammed, H. and T. Rashid^25^ presented the Fox Optimizer (FOX), a unique nature-inspired optimization algorithm that imitates the foraging habits of foxes when pursuing prey in the wild. The performance of the model is assessed using five traditional benchmark functions and benchmark test functions from CEC2019, and the results demonstrate that the FOX performs statistically much better than the comparison algorithms.

Abdullah J.M. and T. Ahmed^26^ proposed the fitness-dependent optimizer (FDO), a revolutionary swarm intelligent algorithm FDO uniquely computes velocity; it leverages the problem fitness function value to generate weights, which guides the search agents throughout both the exploration and exploitation phases. FDO is evaluated on a set of 19 traditional benchmark test functions, and the results are compared to three well-known algorithms: the genetic algorithm (GA), the dragonfly algorithm (DA), and PSO.

The FDO results demonstrate superior performance in the majority of cases. Mohammadi, M. et al.^27^ suggested the donkey and smuggler optimization algorithm (DSO). The DSO was inspired by how donkeys look for things. The experimental results were divided into two sections. First, we employed benchmark test functions to compare the algorithm’s performance to that of the most well-known and cutting-edge algorithms. Second, three real-world applications—the traveling salesman problem, packet routing, and ambulance routing—are used to adapt and implement the DSO. DSO's experimental results on these real-world situations are quite promising.

Mohammed, H.M., S.U. Umar, and T.A. Rashid^28^ gave a systematic and meta-analysis study of WOA to assist researchers in using it in various domains or hybridizing it with other prevalent algorithms. WOA modifications and hybridizations’ statistical outcomes are established and compared to the most prevalent optimization techniques and WOA. According to the survey results, WOA outperforms other common algorithms in terms of convergence speed and balancing exploration and exploitation.

Abdulhameed S. and T.A. Rashid^29^ introduced a revolutionary metaheuristic child drawing development optimization (CDDO) algorithm inspired by children’s learning behavior and cognitive development that uses the golden ratio to optimize the beauty of their artwork CDDO outperforms the 19 benchmark functions in finding the global optimum solution to optimization issues. Its output is compared to the output of multiple cutting-edge algorithms, including, DE, PSO, GSA, FEP, and WOA. The test results reveal that CDDO is relatively competitive, with a score of 2.8. This demonstrates the CDDO exceptional fortitude in seeking a fresh solution.

Rahman C.M. and T.A. Rashid^30^ suggested a learner performance-based behavior algorithm (LPB), a revolutionary evolutionary algorithm. To demonstrate the proposed algorithm’s accuracy, it is tested against a variety of test functions, including standard benchmark functions, CECC06 2019 test functions, and a real-world case study issue. The suggested algorithm’s results are then compared to the DA, GA, and PSO. The proposed method yielded superior outcomes in the majority of situations and comparable results in others.

Hama Rashid D.N., T.A. Rashid and S. Mirjalili^31^ proposed the ant nesting algorithm (ANA), a revolutionary swarm intelligent algorithm. The algorithm was inspired by Leptothorax ants and simulates the behavior of ants looking for places to deposit grains while constructing a new nest. A comparison study with the particle swarm optimization (PSO), genetic algorithm (GA), five modified versions of PSO, dragonfly algorithm (DA), salp swarm algorithm (SSA), fitness dependent optimizer (FDO), and whale optimization algorithm (WOA) validates the results of the ANA algorithm on 26 well-known test functions. In some test cases, ANA outperforms these popular metaheuristic algorithms and produces competitive results.

## Methods

### The whale optimization algorithm

The whale optimization algorithm (WOA) is a population-based meta-heuristic algorithm simulated by the hunting strategy of humpback whales. When humpback whales locate their prey, they dive twelve meters below the surface before ascending in a spiral of bubbles. Encircling prey, spiral bubble-net feeding maneuver, and prey search are the three primary phases of the location update^32^.

Humpback whales use two methods during exploitation, to alter their positions in the direction of the global optimum: reducing encircling (encircling prey) and helix location updating. They consider the most recent ideal site to be the intended prey (global optimum) (spiral bubble-net feeding maneuver). The mathematical paradigm of the shrinking encircling process is depicted as^32^:

1 $$\left| D \right| = \left| {C \cdot X^{*} \left( t \right) - X\left( t \right)} \right|,$$

2 $$X\left( {t + 1} \right) = X^{*} \left( t \right) - A \cdot D.$$

In which *X* is the location vector, X* represents the best solution found thus far, and if a better solution emerges, it will be updated in each iteration. The variables *t* and |⋅| stand for the present iteration, the utter value operation, and $\cdot$ refers to element-by-element multiplication, respectively. Here, the two parameters $A$ and $C$ are determined by using the following^32^.

3 $$\left| A \right| = 2a \cdot r - \left| a \right|,$$

4 $${\text{and}}\,\,C = 2r,$$

where *r* is a randomized value between [0, 1], a drop linearly from 2 to 0 throughout the course of iterations (during both the exploration and exploitation phases) to enable the achievement of the diminishing conduct that encircles.

A spiral equation utilized to mathematically express the spiral updating position, as^32^.

5 $$X\left( {t + 1} \right) = D^{\prime}{\text{e}}^{bl} \cos \left( {2\pi l} \right) + X^{*} \left( t \right),$$

6 $${\text{where}}\,\,D^{\prime} = \left| {X^{*} \left( t \right) - X\left( t \right)} \right|.$$

In this formula, $D^{\prime}$ denotes the separation between the ith whale, and the optimum solution discovered thus far, *l* is a randomized value in the range [− 1,1], and *b* is a constant used to define the form of a logarithmic spiral. It is important to note that when whales grab their prey, they simultaneously use a spiral-shaped track and a shrinking surrounding. Each method has a 50% chance of being used to mimic this behavior^32^.

7 $$X\left( {t + 1} \right) = \left\{ {\begin{array}{*{20}c} {X^{*} \left( t \right) - A \cdot D\,\,\,\,\,\,\,\,\,\,\,\,\,\,\,\,\,\,\,\,\,\,\,\,\,\,\,\,\,\,\,if\,p < 0.5} \\ {D^{\prime}{\text{e}}^{bl} \cos \left( {2\pi l} \right) + X^{*} \left( t \right)\,\,\,\,\,\,\,\,\,\,if\,p \ge 0.5} \\ \end{array} } \right.,$$

where *p* is a randomized value between 0 and 1. Here, a global search is created to improve the capacity for discovery. Its mathematical model is comparable to Eqs. (5) and (6), with the exception that the search is guided by a randomized search agent instead of the best agent. It is decided whether to update the position through exploration (search for prey) or exploitation (a shrinking encircling mechanism) using the randomized variable $\left| A \right|$ with a value higher than 1 and lower than − 1, as^32^.

8 $$D = \left| {C \cdot X_{rand} - X} \right|,$$

9 $$X\left( {t + 1} \right) = X_{rand} - A \cdot D,$$

where *X*_rand_ in this case is a randomized location vector picked from the current generation.

### Golden jackal optimization

Golden jackal optimization (GJO) is a powerful metaheuristic algorithm that replicates the natural hunting behavior of golden jackals. Males and females hunt together frequently with golden jackals. The three stages of the golden jackal’s hunting habit are looking for the prey and advancing toward it, surrounding and agitating the prey until comes to a stop, and finally swooping down on the prey^33^.

During the startup step, a collection of prey location matrices are randomly distributed. Generated by the following equation^33^.

10 $$Prey = \left[ {\begin{array}{*{20}c} {X_{1,1} } & {X_{1,2} } & \cdots & {X_{1,d} } \\ {X_{2,1} } & {X_{2,2} } & \cdots & {X_{2,d} } \\ \vdots & \vdots & \vdots & \vdots \\ {X_{n,1} } & {X_{n,2} } & \cdots & {X_{n,d} } \\ \end{array} } \right],$$

where $X_{i,j}$ denotes the *j*-th dimension of *i*-th prey, *N* stands for the numeral of prey populations, and *n* for dimensions with *d* variables. The golden jackal's hunt can be mathematically described as follows (|*E*|> 1)^33^:

11 $$X_{1} \left( t \right) = X_{M} \left( t \right) - E \cdot \left| {X_{M} \left( t \right) - rl \cdot prey\left( t \right)} \right|,$$

12 $$X_{2} \left( t \right) = X_{FM} \left( t \right) - E \cdot \left| {X_{FM} \left( t \right) - rl \cdot prey\left( t \right)} \right|,$$

in which *t* denotes the current iteration, *prey(t)* represents the prey’s position vector, $X_{M} \left( t \right)$ denotes the male golden jackal’s location, and $X_{FM} \left( t \right)$ denotes the female’s position. The most recent positions of the male and female golden jackals are $X_{1} \left( t \right)$ and $X_{2} \left( t \right)$, respectively. The prey’s evading energy, *E*, is computed from^33^.

13 $$E = E_{1} * E_{0} ,$$

where *E*_*0*_ is a random value between − 1 and 1, representing the prey's beginning energy, and* E*_*1*_ indicates the decreasing energy of the prey as^33^.

14 $$E_{1} = c_{1} * \left( {1 - t/T} \right).$$

The maximum number of repetitions is *T*, $c_{1}$ is the default steady and is set to 1.5. *E*_*1*_ represents the prey’s diminishing energy from 1.5 to 0 through the iterations. The term $\left| {X_{M} \left( t \right) - rl \cdot prey\left( t \right)} \right|$ in Eqs. (11) and (12) indicates the space among the golden jackal and the prey, as this distance gets subtracted or added to the current position of the jackal according to the evading energy of the prey. The vector $rl$ is randomized integers set by the Lévy flight function. The multiplication of $rl$ and $prey\left( t \right)$ mimics the movement of prey in Lévy movement and can be calculated as^33^.

15 $$rl = 0.05 * LF\left( x \right),$$

where the levy flight function $LF\left( x \right)$ is calculated from^33^.

16 $$LF\left( x \right) = \frac{{0.01 \times \left( {\mu \times \sigma } \right)}}{{\left| {\nu^{1 - \beta } } \right|}}\,;\,\,\sigma = \left( {\frac{{\Gamma \left( {1 + \beta } \right) \times \sin \left( {\pi \beta /2} \right)}}{{\Gamma \left( {\frac{1 + \beta }{2}} \right) \times \beta \times 2^{{\frac{\beta - 1}{2}}} }}} \right)^{1/\beta } ,$$

in which $\mu$ and $\nu$ are arbitrary values inside (0, 1) and $\beta$ is the default constant with the value 1.5. Consequently, the jackal positions are updated by taking the mean of Eq. (11) & Eq. (12), as^33^.

17 $$X\left( {t + 1} \right) = \frac{{X_{1} \left( t \right) + X_{2} \left( t \right)}}{2},$$

as $X\left( {t + 1} \right)$ is the location of the prey that has been updated by the male and female golden jackals, the prey’s evasive energy decreases as a result of the golden jackals’ harassment. The golden jackals' mathematical model of how they surround and eat their victims is as follows (|*E*|≤ 1)^33^.

18 $$X_{1} \left( t \right) = X_{M} \left( t \right) - E \cdot \left| {rl \cdot X_{M} \left( t \right) - prey\left( t \right)} \right|,$$

19 $$X_{2} \left( t \right) = X_{FM} \left( t \right) - E \cdot \left| {rl \cdot X_{FM} \left( t \right) - prey\left( t \right)} \right|.$$

### Proposed method

**Figure 1:**
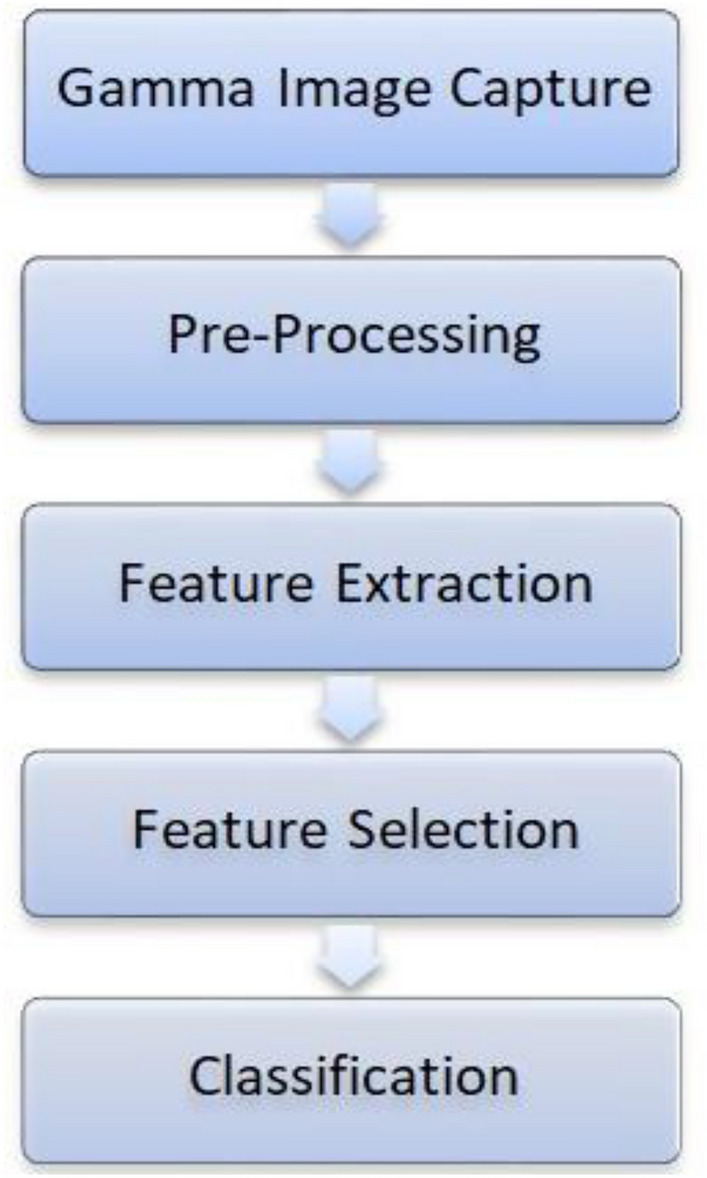
Phases of the proposed method.

Four stages make up the suggested method: pre-processing, feature extraction, feature selection, and classification. First, the input image of the bone gamma-camera scanned was segmented. The features retrieved from the segmented image are then utilized to forecast the classification of the bone state using machine learning. The next subsections describe the feature extraction and feature selection phases in depth. Figure 1 shows the phases of the proposed method.

#### Feature extraction

**Figure 2:**
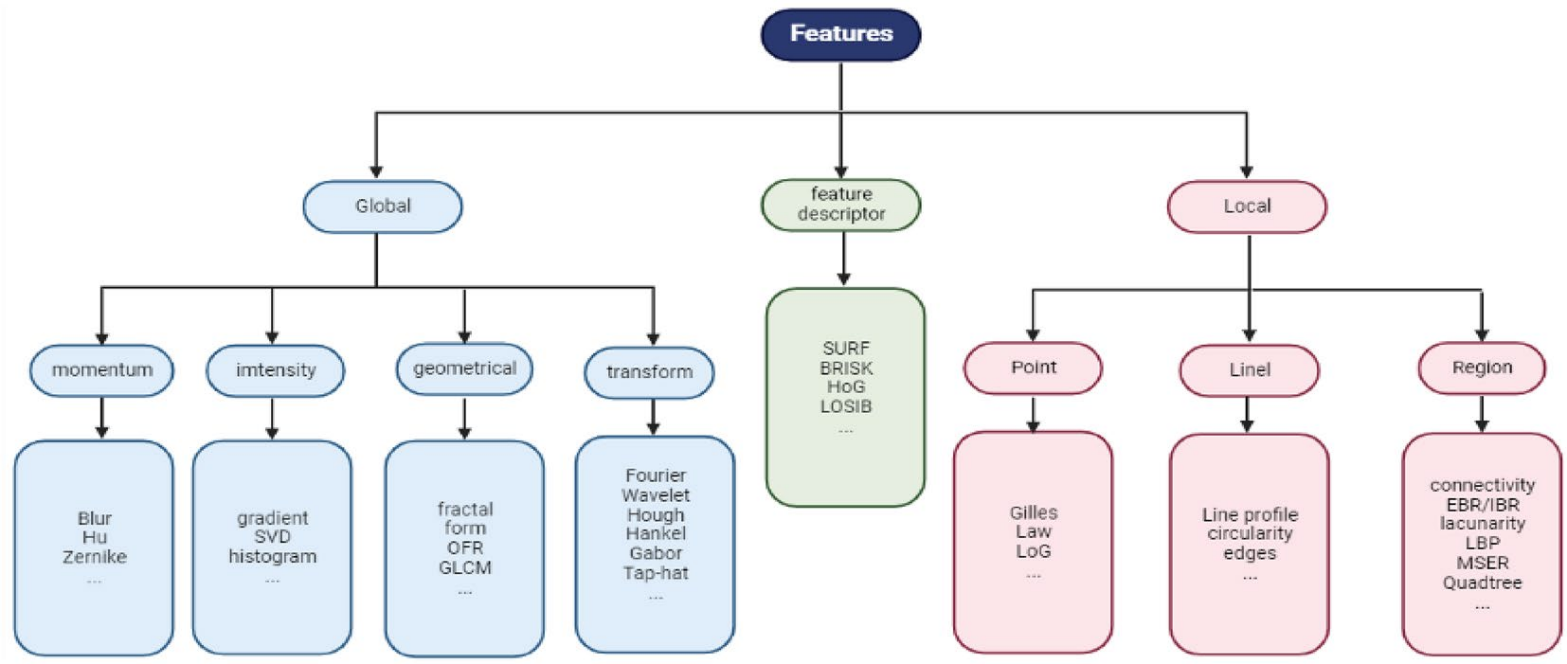
The type of features used in this study.

We provide a variety of algorithms for feature extraction from a medical image. As shown in Fig. 2, they could be nearly categorized into three groups: local features, global features, and feature descriptors^34^.

##### Global features

Global features consider the entire image composition. Because they can describe a wide range of traits, they are further classified into the subcategories listed below.

- Moment features: In this denomination, features are obtained from various image moments using the HU moments and Zernike algorithms, as well as affine moments and blur.
- Intensity features: These features are dependent on the variety of input image intensities, and the algorithms utilized are various intensity mensuration, image gradients, histograms, and image singular value decomposition.
- Geometrical features: The structural qualities of an image are described by features in this category. An example, Fractal dimension characteristics, for example, quantify how self-alike structures inside a picture are, Other geometrical features are dependent on run length, form factor, or gray-level co-occurrence matrices (GLCM).
- Transformation features: An image can go through a variety of transformations, and features are therefore calculated from the altered image, the transformation features encompass (discrete cosine, Fourier, distance, Hankel, top-hat, Hough, various unitary) transform, skeletonization, and Gabor filters.

##### Local features

Local features, as opposed to global features. The structures of tinier-picture regions are reflected. Depending on the kind of local structure they are extracted from, local features can be divided into three subclasses.

- Point features: The algorithms utilized are Laplacian of Gaussian (LoG), Gilles key points, and Law features, these features are based on various key point kinds and their characteristics, such as the quantity or distribution of detected points.
- Line features: This category’s features are calculated using lines in the image and information are taken from them. using characteristics of linear structures, edges of detected objects, corners, and circles, as well as measurements generated from line profiles.
- Region features: The initial set is derived from certain image regions. Regions could be identified objects or patterns in an image, or they can be predetermined portions, Smaller sub-images, for example. The region feature algorithms included are based on the connection of detected objects, saliency, lacunarity, edge-based region (EBR)/intensity extrema-based region (IBR), maximally stable extremal region (MSER), local binary pattern (LBP), and sub-images based on quadtree decomposition or sector division.

The third denomination of features is region covariance descriptors, locally oriented statistics information booster (LOSIB), and speeded-up robust features (SURF), which can express information on both global and/or local image attributes.

#### Suggested feature selection algorithm

Feature extraction was followed by feature selection (FS). This is a critical step since a well-chosen set of differentiating features enables the creation of a high-accuracy classifier. The suggested feature selection algorithm is integrated with two algorithms: Golden jackal optimization (GJO) and optimization algorithm (WOA).

The suggested FS algorithm is named GJOW, which compiles the GJO and WOA algorithms. The fundamental structure of the GJO algorithm is improved by enhancing the population’s position update phase. This modification integrates the WOA’s update mechanism into the major structure of the GJO. This integration gives the GJO more flexibility in discovering the population and ensuring its variability, as well as quickly reaching the optimum values. The suggested GJOW’s initial step is to determine the parameters and create the population, which symbolizes a group of solutions to the issue at hand (feature selection).

The efficiency of each solution is then assessed by calculating its fitness function and selecting the best one. The suggested GJOW algorithm’s subsequent phase involves updating the current population using either the GJO or WOA algorithm, relying on the effectiveness of the fitness function, the GJO is employed in this case if the fitness function probability for the current solution is larger than 0.15; otherwise, the WOA is utilized. After computing the fitness function for each solution, the best one is selected after updating the population. If the stop conditions are met, the next step is to return to the best solution; if not, repeat the previous steps from calculating the probability to the end. In the paragraphs that follow, these steps are covered in more detail.

The GJOW algorithm begins by specifying the initial parameters for the GJO and the WOA. Next, the GJO produces a random population X of size N in dimension D, after which the GJO measures the fitness of every solution $x_{i}$, $i = 1,2, \cdots ,N$. However, before determining the objective function, every solution $x_{i}$ is transformed into a binary vector (consisting exclusively of *1's* and *0*’*s*) based on the value of a randomized threshold $\varepsilon \in \left[ {0,1} \right]$ using the following equation:

20 $$x_{i} \left( {t + 1} \right) = \left\{ {\begin{array}{*{20}c} {1\,\,\,\,\,\,\,\,\,\,\,\,\,\,\,if\,x_{1} > \varepsilon } \\ {0\,\,\,\,\,\,\,\,\,\,\,\,\,otherwise} \\ \end{array} } \right..$$

The chosen features are therefore represented by only the $x_{i}$ elements that match *1’s*. Furthermore, the other elements are discarded because they exemplify irrelevant features. The objective function for each $x_{i}$ in the equation must be computed in the following step, as:

21 $$f\left( {x_{i} \left( t \right)} \right) = \varepsilon Ex_{i} \left( t \right) + \left( {1 - \varepsilon } \right)\frac{{\left| {x_{i} \left( t \right)} \right|}}{\left| C \right|},$$

where $Ex_{i} \left( t \right)$ is the classification error of the effective classifier. The second term corresponds to the KNN classifier that gives the number of the selected features. The parameter $\varepsilon \in \left[ {0,1} \right]$ is employed to strike a compromise between the number of picked features and the classification error. The probability of each fitness function $P_{ROI}$ is then computed as:

21 $$P_{ROI} = \frac{{f_{i} }}{{\sum\nolimits_{i = 1}^{N} {f_{i} } }}.$$

The GJO or WOA will be used to update the existing solution $x_{i}$ in accordance with the $P_{ROI}$ value. For instance, the WOA algorithm is used as described in Sect. “The literature of ML for bone metastasis detection”. if $P_{ROI} > 0.15$; otherwise, the GJO algorithm is used as described in Sect. “The literature of application of MH techniques”.

**Figure 3:**
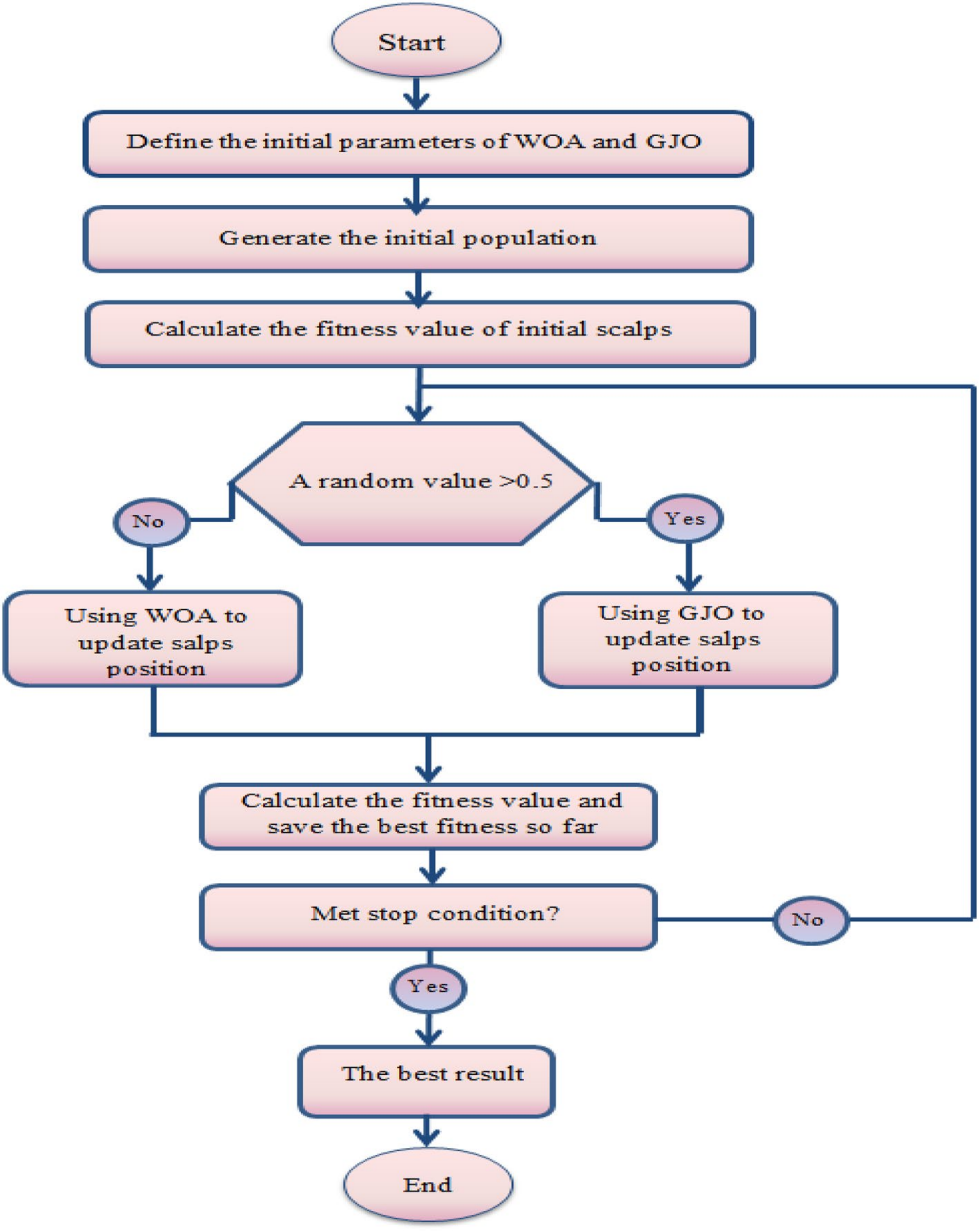
The main structure of the proposed algorithm.

For each updated solution, the fitness function is calculated, and the top update is made. This sequence is repeated until the stopping requirement is satisfied (the suggested GJOW algorithm uses the maximum number of iterations as a stopping condition). Figure 3 shows the main structure of the proposed algorithm.

Time complexity of the developed method depends on some factors invluding number of iterations, solutions, and features. So, we can formulated the time complexity of developed method as:

22 $$O\left(GJOW\right)=O\left(N\times D+t\times \left({K}_{1}\times D+{K}_{2}\times D\right)\right),$$

where ${K}_{1}$ and ${K}_{2}$ refers to number of solutions updated using WOA and GJO, respectively. $D$ stands for the features and $T$ number of iterations.

## Results

### Data description

We used a database in order to assess the efficacy of our suggested algorithm, we chose 581 patients from this database (362 abnormal and 219 normal) women and men aged between 3 and 90 years who had skeletal scintigraphy imaging due to probable bone metastatic illness. The outcome is an image with two dimensions: an anterior and a posterior.

**Figure 4:**
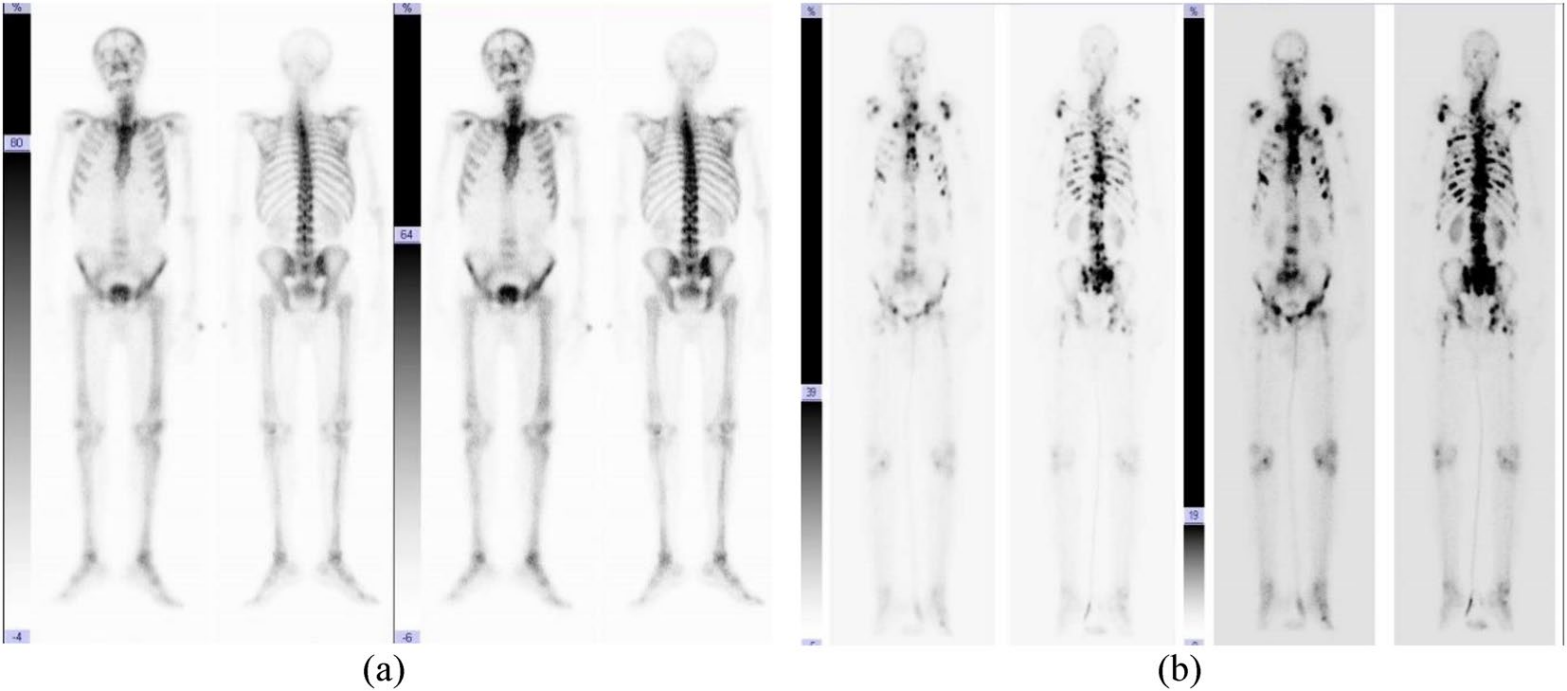
Skeletal scintigraphy image with (**a**) normal (**b**) abnormal.

Each image is broken into two sections of varying brightness, for example, the first image has two parts that range from 80 to 64%, and the second from 39 to 19%, Fig. 4 shows an illustration of skeletal scintigraphy pictures. In addition, we used 23 UCI machine-learning datasets as benchmark to evaluate the performance of developed FS model.

### Parameter setting and performance metrics

The results of the GJOW are compared to seven methods: GJO, BPSO, WOA, EO, HHO, SSA, and ASO. The parameters of each algorithm are assigned according to the original implementation as mentioned in Table 1. In addition, the common parameters such as the number of solutions and iterations are set to 15 and 100, respectively.

**Table 1:** The parameter value of each algorithm.

Algorithm	Parameter	Value
GJO	C1	1.5
BPSO	W, c1, c2	W = 2, C1 = 2, C2 = 2,
WOA	b	1
EO	GP, a1, a2	GP = 0.5; a1 = 2; a2 = 1
HHO	E, beta	E€[0.2], beta = 1.5
SSA	C1, C2	C1€ [01], C2€ [01]
ASO	Alpha, beta	Alpha = 50, beta = 0.2
GJOW	C1, b	C1 = 1.5, b = 1

In addition, seven performance measures are applied: the standard deviation, average (Avg), maximum (Max), and minimum (Min) of the value of the objective function. Moreover, the accuracy (ACC), sensitivity (SEN), and specificity (SPE) are also estimated.

### Results and discussion of benchmark datasets

**Figure 5:**
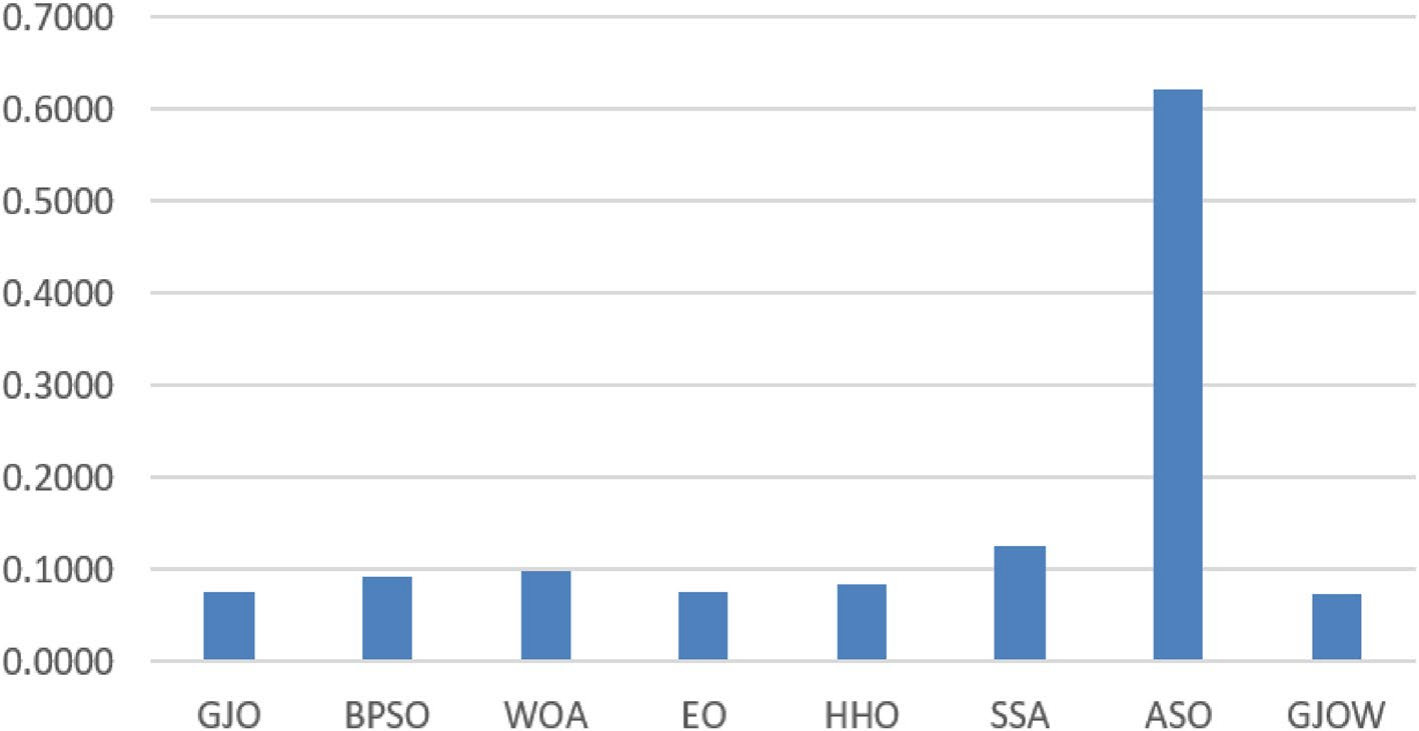
Average of the objective function for the datasets.

The comparative results of the developed method are given in Tables 2, 3, 4, 5, 6, 7, 8 and 9. Table 2 presents the average of the objective function for the GJOW and the other methods for twenty-three datasets. In Table 2, the GJOW obtained the top outcomes in 6 of 23 datasets (i.e. Brain_T91, IonosphereEW5, KrvskpEW4, leuk1, and PenglungEW3) whereas the GJOW, GJO, and BPSO showed the best results in 5 datasets; these results ranked the GJOW as the best method amongst the compared algorithms. The BPSO obtained the second grade by getting the best average in four datasets (i.e. Breastcancer4, Lymphography3, Tic-tac-toe3, and base_Vote3). The GJO was ranked third by getting the best average in four datasets (i.e. BreastEW12, base_HeartEW2, SpectEW2, and WaveformEW9). The EO and WOA showed promising outcomes and came in fourth and fifth respectively. Whereas the remaining methods were sorted as follows: HHO, SSA, and ASO, respectively. Figure 5 illustrates the objective functions’ average for the datasets. In this figure, the algorithms names are listed in x-axis, while the average values of the objective function are listed in y-axis. The shorter bar (i.e. GJOW) is for the better algorithm whereas, the longer bar indicates the worst algorithm.

**Table 2:** Average of fitness value.

AVG	GJO	BPSO	WOA	EO	HHO	SSA	ASO	GJOW
base_Brain_T21	0.0180	0.0870	**0.0014**	0.0151	0.0053	0.2107	0.6139	0.0032
base_Brain_T91	0.0998	0.2264	0.1094	0.0859	0.1059	0.3054	0.6516	**0.0212**
base_Breastcancer4	0.0586	**0.0544**	0.0622	0.0593	0.0561	0.0630	0.6790	0.0615
base_BreastEW	0.0459	0.0524	0.0702	**0.0422**	0.0617	0.0886	0.6403	0.0548
base_BreastEW12	**0.0317**	0.0366	0.0442	0.0327	0.0402	0.0554	0.5787	0.0324
base_CongressEW1	**0.0228**	**0.0228**	0.0238	**0.0228**	0.0241	0.0551	0.6334	0.0241
base_Exactly	0.0470	**0.0462**	0.0740	0.0470	0.0508	0.0694	0.6588	**0.0462**
base_Exactly2	**0.1877**	**0.1877**	**0.1877**	0.1920	**0.1877**	0.2172	0.6064	0.2152
base_Exactly11	**0.0462**	0.0925	0.1639	**0.0462**	0.0492	0.0980	0.5486	**0.0462**
base_HeartEW2	**0.1219**	0.1233	0.1390	0.1262	0.1258	0.1481	0.5610	0.1312
base_IonosphereEW5	0.0370	0.0482	0.0625	0.0355	0.0590	0.0847	0.6247	**0.0328**
base_KrvskpEW4	0.0530	0.0638	0.0813	0.0509	0.0752	0.0834	0.6624	**0.0507**
base_leuk1	0.0332	0.0961	0.0171	0.0144	0.0079	0.1185	0.5950	**0.0023**
base_Lymphography3	0.1250	**0.1116**	0.1564	0.1142	0.1274	0.1440	0.6068	0.1285
base_M-of-n3	**0.0462**	**0.0462**	0.0951	0.0470	0.0515	0.0620	0.7489	**0.0462**
base_PenglungEW3	0.0169	0.0492	0.0244	0.0250	0.0474	0.1201	0.5931	**0.0142**
base_SonarEW2	0.0509	0.0865	0.1117	0.0628	0.0900	0.1259	0.5998	**0.0509**
base_SpectEW2	**0.1056**	0.1070	0.1347	0.1082	0.1217	0.1377	0.5740	0.1103
base_Tic-tac-toe3	0.2424	**0.2316**	0.2631	0.2388	0.2354	0.2397	0.6162	0.2455
base_Vote3	0.0671	**0.0597**	0.0822	0.0638	0.0728	0.0911	0.5797	0.0811
base_WaveformEW9	**0.2372**	0.2610	0.2927	0.2388	0.2744	0.2820	0.5863	0.2514
base_WineEW1	0.0510	**0.0393**	0.0675	0.0425	0.0456	0.0595	0.6587	**0.0393**
Zoo	**0.0188**	**0.0188**	0.0244	0.0219	0.0225	0.0306	0.6714	**0.0188**

Best values are in bold.

**Table 3:** STD results for fitness value.

STD	GJO	BPSO	WOA	EO	HHO	SSA	ASO	GJOW
base_Brain_T21	0.0010	0.0492	0.0023	0.0046	0.0150	0.0508	0.1656	**0.0006**
base_Brain_T91	0.0833	0.0387	0.1010	0.0708	0.0698	**0.0018**	0.1634	0.0375
base_Breastcancer4	0.0059	**0.0000**	0.0041	0.0019	0.0028	0.0046	0.1963	0.0049
base_BreastEW	0.0076	0.0085	0.0121	0.0094	0.0086	0.0106	0.1440	**0.0072**
base_BreastEW12	0.0064	**0.0034**	0.0073	0.0042	0.0054	0.0065	0.0732	0.0063
base_CongressEW1	**0.0000**	**0.0000**	0.0033	**0.0000**	0.0027	0.0100	0.1853	0.0027
base_Exactly	0.0024	**0.0000**	0.0557	0.0024	0.0039	0.0275	0.1218	**0.0000**
base_Exactly2	**0.0000**	**0.0000**	**0.0000**	0.0088	**0.0000**	0.0245	0.1128	0.0445
base_Exactly11	**0.0000**	0.0976	0.0968	**0.0000**	0.0039	0.0678	0.0903	**0.0000**
base_HeartEW2	**0.0032**	0.0040	0.0065	0.0097	0.0057	0.0183	0.1083	0.0089
base_IonosphereEW5	0.0109	**0.0062**	0.0179	0.0113	0.0082	0.0115	0.1733	0.0095
base_KrvskpEW4	0.0059	**0.0019**	0.0142	0.0028	0.0077	0.0076	0.1348	0.0035
base_leuk1	0.0289	0.0248	0.0265	0.0182	0.0128	**0.0003**	0.1696	0.0007
base_Lymphography3	**0.0120**	0.0129	0.0150	0.0169	0.0174	0.0164	0.1313	0.0229
base_M-of-n3	**0.0000**	**0.0000**	0.0453	0.0024	0.0052	0.0116	0.2193	**0.0000**
base_PenglungEW3	0.0189	0.0184	0.0292	0.0251	0.0294	**0.0031**	0.1166	0.0203
base_SonarEW2	0.0190	**0.0109**	0.0314	0.0177	0.0225	0.0173	0.1166	0.0192
base_SpectEW2	0.0082	0.0073	0.0151	0.0081	0.0097	0.0095	0.1184	**0.0047**
base_Tic-tac-toe3	0.0110	**0.0030**	0.0153	0.0106	0.0050	0.0102	0.1282	0.0081
base_Vote3	0.0110	**0.0070**	0.0096	0.0095	0.0083	0.0135	0.1382	0.0163
base_WaveformEW9	0.0073	0.0063	0.0172	**0.0059**	0.0099	0.0121	0.1941	0.0148
base_WineEW1	0.0113	**0.0024**	0.0195	0.0080	0.0089	0.0138	0.1561	0.0105
Zoo	**0.0000**	**0.0000**	0.0062	0.0044	0.0052	0.0046	0.1125	**0.0000**

Best values are in bold.

**Table 4:** Max results of fitness value.

Max	GJO	BPSO	WOA	EO	HHO	SSA	ASO	GJOW
base_Brain_T21	0.0193	0.1487	0.0076	0.0232	0.0479	0.2602	0.9226	**0.0046**
base_Brain_T91	0.2631	0.2933	0.2789	0.2046	0.2267	0.3096	0.9089	**0.1049**
base_Breastcancer4	0.0719	**0.0544**	0.0719	0.0608	0.0608	0.0684	1.1165	0.0719
base_BreastEW	**0.0537**	0.0649	0.0840	0.0604	0.0761	0.1028	0.9199	0.0670
base_BreastEW12	0.0479	0.0412	0.0561	0.0425	0.0504	0.0646	0.6988	**0.0404**
base_CongressEW1	**0.0228**	**0.0228**	0.0332	**0.0228**	0.0291	0.0728	0.8430	0.0291
base_Exactly	0.0538	**0.0462**	0.2299	0.0538	0.0538	0.1457	0.8248	**0.0462**
base_Exactly2	**0.1877**	**0.1877**	**0.1877**	0.2153	**0.1877**	0.2635	0.8009	0.2865
base_Exactly11	**0.0462**	0.2777	0.2777	**0.0462**	0.0538	0.2601	0.6571	**0.0462**
base_HeartEW2	0.1308	**0.1295**	0.1410	0.1462	0.1397	0.1795	0.7535	0.1410
base_IonosphereEW5	**0.0489**	0.0548	0.0840	0.0577	0.0733	0.1027	0.9742	0.0577
base_KrvskpEW4	0.0630	0.0668	0.1087	0.0571	0.0907	0.0907	0.9575	**0.0558**
base_leuk1	0.0753	0.1080	0.0610	0.0656	0.0337	0.1189	0.9110	**0.0039**
base_Lymphography3	0.1511	**0.1320**	0.1778	0.1344	0.1511	0.1622	0.8256	0.1700
base_M-of-n3	**0.0462**	**0.0462**	0.1978	0.0538	0.0615	0.0827	1.1774	**0.0462**
base_PenglungEW3	0.0705	0.1012	0.0695	0.0723	0.0840	0.1255	0.7382	0.0717
base_SonarEW2	0.0812	0.1043	0.1588	0.0895	0.1190	0.1440	0.7482	**0.0745**
base_SpectEW2	**0.1152**	**0.1152**	0.1561	0.1197	0.1318	0.1576	0.7655	0.1182
base_Tic-tac-toe3	0.2571	**0.2401**	0.2858	0.2571	**0.2401**	0.2571	0.8844	0.2571
base_Vote3	0.0912	**0.0700**	0.0962	0.0763	0.0825	0.1075	0.8263	0.1075
base_WaveformEW9	**0.2478**	0.2655	0.3148	0.2536	0.2834	0.2972	0.9420	0.2821
base_WineEW1	0.0635	**0.0462**	0.0981	0.0635	0.0635	0.0885	0.9887	0.0635
Zoo	**0.0188**	**0.0188**	0.0375	0.0312	0.0312	0.0375	0.8214	**0.0188**

Best values are in bold.

**Table 5:** Min results of fitness value.

Min	GJO	BPSO	WOA	EO	HHO	SSA	ASO	GJOW
base_Brain_T21	0.0168	0.0483	0.0001	0.0097	**0.0000**	0.1582	0.3928	0.0027
base_Brain_T91	0.0173	0.1619	**0.0001**	0.0182	0.0006	0.3035	0.4256	0.0030
base_Breastcancer4	**0.0544**	**0.0544**	0.0590	**0.0544**	**0.0544**	**0.0544**	0.4520	**0.0544**
base_BreastEW	**0.0279**	0.0391	0.0525	0.0312	0.0504	0.0670	0.4804	0.0458
base_BreastEW12	0.0246	0.0312	0.0358	0.0279	0.0312	0.0433	0.4735	**0.0212**
base_CongressEW1	**0.0228**	**0.0228**	**0.0228**	**0.0228**	**0.0228**	0.0438	0.2577	**0.0228**
base_Exactly	**0.0462**	**0.0462**	**0.0462**	**0.0462**	**0.0462**	0.0538	0.5100	**0.0462**
base_Exactly2	**0.1877**	**0.1877**	**0.1877**	**0.1877**	**0.1877**	0.1954	0.4668	**0.1877**
base_Exactly11	**0.0462**	**0.0462**	**0.0462**	**0.0462**	**0.0462**	**0.0462**	0.4022	**0.0462**
base_HeartEW2	**0.1205**	**0.1205**	**0.1205**	**0.1205**	0.1218	**0.1205**	0.4116	**0.1205**
base_IonosphereEW5	**0.0176**	0.0362	0.0371	0.0235	0.0459	0.0656	0.4458	0.0235
base_KrvskpEW4	**0.0475**	0.0614	0.0657	**0.0475**	0.0641	0.0682	0.5107	**0.0475**
base_leuk1	0.0140	0.0489	0.0002	0.0048	**0.0001**	0.1182	0.3855	0.0017
base_Lymphography3	0.1100	**0.0911**	0.1367	**0.0911**	0.0933	0.1121	0.3862	**0.0911**
base_M-of-n3	**0.0462**	**0.0462**	0.0538	**0.0462**	**0.0462**	**0.0462**	0.3239	**0.0462**
base_PenglungEW3	0.0074	0.0388	**0.0018**	0.0089	0.0114	0.1166	0.3561	0.0052
base_SonarEW2	0.0283	0.0614	0.0779	0.0350	0.0531	0.0962	0.3538	**0.0200**
base_SpectEW2	**0.0939**	0.0985	0.1061	**0.0939**	0.1030	0.1242	0.4088	0.1061
base_Tic-tac-toe3	**0.2307**	**0.2307**	0.2401	**0.2307**	**0.2307**	**0.2307**	0.4380	0.2401
base_Vote3	**0.0525**	**0.0525**	0.0700	**0.0525**	**0.0525**	0.0650	0.3679	0.0612
base_WaveformEW9	**0.2235**	0.2458	0.2678	0.2304	0.2526	0.2618	0.3715	0.2278
base_WineEW1	**0.0385**	**0.0385**	**0.0385**	**0.0385**	**0.0385**	0.0462	0.5208	**0.0385**
Zoo	**0.0188**	**0.0188**	**0.0188**	**0.0188**	**0.0188**	0.0250	0.5036	**0.0188**

Best values are in bold.

**Table 6:** Accuracy results for each FS method.

ACC	GJO	BPSO	WOA	EO	HHO	SSA	ASO	GJOW
base_Brain_T21	0.900	0.900	0.900	0.900	0.900	0.900	0.900	**1.000**
base_Brain_T91	**1.000**	0.778	**1.000**	0.818	0.875	0.636	0.500	**1.000**
base_Breastcancer4	0.957	0.957	0.950	0.957	0.957	0.950	0.950	**0.971**
base_BreastEW	0.974	0.983	0.939	0.991	0.956	0.974	0.912	**1.000**
base_BreastEW12	0.983	0.974	0.965	0.991	0.965	0.974	0.930	**1.000**
base_CongressEW1	0.977	0.977	0.966	0.977	0.977	0.954	0.908	**1.000**
base_Exactly	**1.000**	**1.000**	**1.000**	**1.000**	**1.000**	**1.000**	0.745	**1.000**
base_Exactly2	0.775	0.780	0.780	0.800	0.780	0.760	0.685	**0.815**
base_Exactly11	**1.000**	**1.000**	0.740	**1.000**	**1.000**	**1.000**	0.715	**1.000**
base_HeartEW2	0.907	0.889	0.833	0.907	0.907	0.889	0.870	**0.972**
base_IonosphereEW5	0.972	0.958	0.958	0.986	0.944	0.930	0.845	**1.000**
base_KrvskpEW4	0.969	0.977	0.955	0.978	0.966	0.969	0.916	**0.988**
base_leuk1	**1.000**	0.933	**1.000**	**1.000**	**1.000**	0.933	0.867	**1.000**
base_Lymphography3	0.967	0.967	0.867	0.967	0.967	0.967	0.833	**1.000**
base_M-of-n3	**1.000**	**1.000**	0.935	**1.000**	**1.000**	0.950	0.830	**1.000**
base_PenglungEW3	**1.000**	0.933	0.933	0.933	0.933	0.933	0.800	**1.000**
base_SonarEW2	**1.000**	0.976	**1.000**	**1.000**	0.952	0.929	0.810	**1.000**
base_SpectEW2	0.889	**0.926**	0.796	**0.926**	**0.926**	0.889	0.833	**0.926**
base_Tic-tac-toe3	0.802	0.802	0.823	0.802	0.807	0.818	0.740	**0.885**
base_Vote3	0.983	**1.000**	0.983	0.983	0.983	0.983	0.917	**1.000**
base_WaveformEW9	0.751	0.753	0.738	0.749	0.750	0.725	0.710	**0.778**
base_WineEW1	**1.000**	**1.000**	0.972	**1.000**	**1.000**	**1.000**	0.917	**1.000**
Zoo	**1.000**	**1.000**	**1.000**	**1.000**	**1.000**	**1.000**	0.952	**1.000**

Best values are in bold.

**Table 7:** Sensitivity results of each FS method.

SEN	GJO	BPSO	WOA	EO	HHO	SSA	ASO	GJOW
base_Brain_T21	**1.0000**	**1.0000**	**1.0000**	**1.0000**	**1.0000**	**1.0000**	**1.0000**	**1.0000**
base_Brain_T91	**0.5000**	**0.5000**	**0.5000**	0.0000	**0.5000**	**0.5000**	0.0000	**0.5000**
base_Breastcancer4	0.9556	0.9556	**0.9667**	0.9556	0.9556	**0.9667**	**0.9667**	0.9639
base_BreastEW	0.9855	**1.0000**	0.9710	**1.0000**	**1.0000**	**1.0000**	0.9275	**1.0000**
base_BreastEW12	**1.0000**	**1.0000**	0.9706	**1.0000**	0.9853	0.9853	0.9559	**1.0000**
base_CongressEW1	0.9643	0.9643	0.9464	0.9643	0.9643	0.9286	0.8750	**1.0000**
base_Exactly	**1.0000**	**1.0000**	**1.0000**	**1.0000**	**1.0000**	**1.0000**	0.8188	**1.0000**
base_Exactly2	0.8590	**1.0000**	**1.0000**	0.8782	**1.0000**	0.8333	0.8141	0.8859
base_Exactly11	**1.0000**	**1.0000**	**1.0000**	**1.0000**	**1.0000**	**1.0000**	0.7432	**1.0000**
base_HeartEW2	0.9459	0.8919	0.8378	0.9459	0.9189	0.9189	0.8649	**0.9750**
base_IonosphereEW5	**1.0000**	0.9767	0.9767	**1.0000**	0.9535	**1.0000**	**1.0000**	**1.0000**
base_KrvskpEW4	0.9612	0.9582	0.9403	0.9582	0.9642	0.9672	0.8925	**0.9777**
base_leuk1	**1.0000**	0.8889	**1.0000**	**1.0000**	**1.0000**	0.8889	0.7778	**1.0000**
base_Lymphography3	**1.0000**	**1.0000**	0.8000	**1.0000**	0.9333	0.8267	0.8667	**1.0000**
base_M-of-n3	**1.0000**	**1.0000**	0.9130	**1.0000**	**1.0000**	0.9710	0.8406	**1.0000**
base_PenglungEW3	**1.0000**	**1.0000**	**1.0000**	**1.0000**	**1.0000**	**1.0000**	**1.0000**	**1.0000**
base_SonarEW2	**1.0000**	0.9565	**1.0000**	**1.0000**	0.9130	0.9565	0.8261	**1.0000**
base_SpectEW2	0.8182	0.8182	0.0000	0.8182	0.7273	**0.9091**	0.7273	0.8889
base_Tic-tac-toe3	**0.9412**	**0.9412**	0.8992	**0.9412**	0.8571	0.8824	0.9076	0.9308
base_Vote3	0.9545	**1.0000**	0.9545	0.9545	0.9545	**1.0000**	0.9091	**1.0000**
base_WaveformEW9	0.7865	0.7573	0.6930	0.7573	0.7281	0.7047	0.6696	**0.7884**
base_WineEW1	**1.0000**	**1.0000**	0.9000	**1.0000**	**1.0000**	**1.0000**	**1.0000**	**1.0000**
Zoo	**1.0000**	**1.0000**	0.8750	0.8750	**1.0000**	**1.0000**	**1.0000**	**1.0000**

Best values are in bold.

**Table 8:** Specificity results of each FS method.

SPE	GJO	BPSO	WOA	EO	HHO	SSA	ASO	GJOW
base_Brain_T21	**1.0000**	**1.0000**	**1.0000**	**1.0000**	**1.0000**	**1.0000**	**1.0000**	**1.0000**
base_Brain_T91	0.7000	0.7000	0.4000	**1.0000**	0.7000	0.9000	**1.0000**	0.8000
base_Breastcancer4	0.9600	0.9600	0.9200	0.9600	0.9600	0.9200	0.9200	**0.9825**
base_BreastEW	0.9556	0.9556	0.8889	0.9778	0.8889	0.9333	0.8889	**1.0000**
base_BreastEW12	0.9559	0.9348	0.9565	0.9783	0.9348	0.9565	0.8913	**1.0000**
base_CongressEW1	**1.0000**	**1.0000**	**1.0000**	**1.0000**	**1.0000**	**1.0000**	**1.0000**	**1.0000**
base_Exactly	**1.0000**	**1.0000**	**1.0000**	**1.0000**	**1.0000**	**1.0000**	0.5806	**1.0000**
base_Exactly2	0.4773	0.0000	0.0000	0.5227	0.0000	0.5000	0.2273	**0.6078**
base_Exactly11	**1.0000**	**1.0000**	0.0000	**1.0000**	**1.0000**	**1.0000**	**1.0000**	**1.0000**
base_HeartEW2	0.8235	0.8824	0.8235	0.8235	0.8824	0.8235	0.8824	**0.9677**
base_IonosphereEW5	0.9286	0.9286	0.9286	0.9643	0.9286	0.8214	0.6071	**1.0000**
base_KrvskpEW4	0.9770	0.9967	0.9705	**1.0000**	0.9672	0.9705	0.9410	0.9969
base_leuk1	**1.0000**	**1.0000**	**1.0000**	**1.0000**	**1.0000**	**1.0000**	**1.0000**	**1.0000**
base_Lymphography3	0.9333	0.9333	0.9333	0.9333	**1.0000**	**1.0000**	0.8000	0.9375
base_M-of-n3	**1.0000**	**1.0000**	0.9466	**1.0000**	**1.0000**	0.9389	0.8244	**1.0000**
base_PenglungEW3	**1.0000**	**1.0000**	**1.0000**	**1.0000**	**1.0000**	**1.0000**	**1.0000**	**1.0000**
base_SonarEW2	**1.0000**	**1.0000**	**1.0000**	**1.0000**	**1.0000**	0.8947	0.7895	**1.0000**
base_SpectEW2	0.9070	0.9535	**1.0000**	0.9535	0.9767	0.8837	0.8605	0.9333
base_Tic-tac-toe3	0.5753	0.5753	0.6986	0.5753	0.7260	0.7123	0.4658	**0.7903**
base_Vote3	**1.0000**	**1.0000**	**1.0000**	**1.0000**	**1.0000**	0.9737	0.9211	**1.0000**
base_WaveformEW9	0.8647	0.8480	0.8678	0.8647	0.8739	0.8480	0.8480	**0.8748**
base_WineEW1	**1.0000**	**1.0000**	**1.0000**	**1.0000**	**1.0000**	**1.0000**	0.9231	**1.0000**
Zoo	**1.0000**	0.9231	0.9231	0.9231	**1.0000**	**1.0000**	**1.0000**	**1.0000**

Best values are in bold.

**Table 9:** Statistical results of each FS method.

Measure	GJO	BPSO	WOA	EO	HHO	SSA	ASO	GJOW
Accuracy	5.24	4.96	3.59	5.48	4.70	3.65	1.26	**7.13**
Sensitivity	5.24	4.98	3.46	4.93	4.35	4.48	2.50	**6.07**
Specificity	4.57	4.39	3.93	5.22	4.87	4.09	2.76	**6.17**

Best values are in bold.

Table 3 shows the standard division results for the methods. The BPSO was ranked first, it showed the smallest value in most datasets. The suggested GJOW method got acceptable standard division results in most cases.

The CHCLPSO, on the other hand, displayed the lowest STD values, followed by lshade and LSHADE-SPACMA. After the EO, HHO, SSA, and WOA, the GJO came in third place. The ASO approach revealed the poorest values for a standard division.

Moreover, Table 4 displays the objective function’s worst values. In this table, the BPSO and suggested GJOW showed the best results in most datasets and were ranked first and second, respectively. The GJO showed the third-best values followed by EO, HHO, WOA, and SSA. The worst values were shown by ASO.

Table 5 records the best outcome of the objective function for all methods. In this regard, the suggested GJOW showed the minimum values in comparison to the other algorithms, it achieved the best standards in most cases. The GJO showed the second-best results whereas, the EO came in the third rank followed by BPSO, HHO, WOA, and SSA.

**Figure 6:**
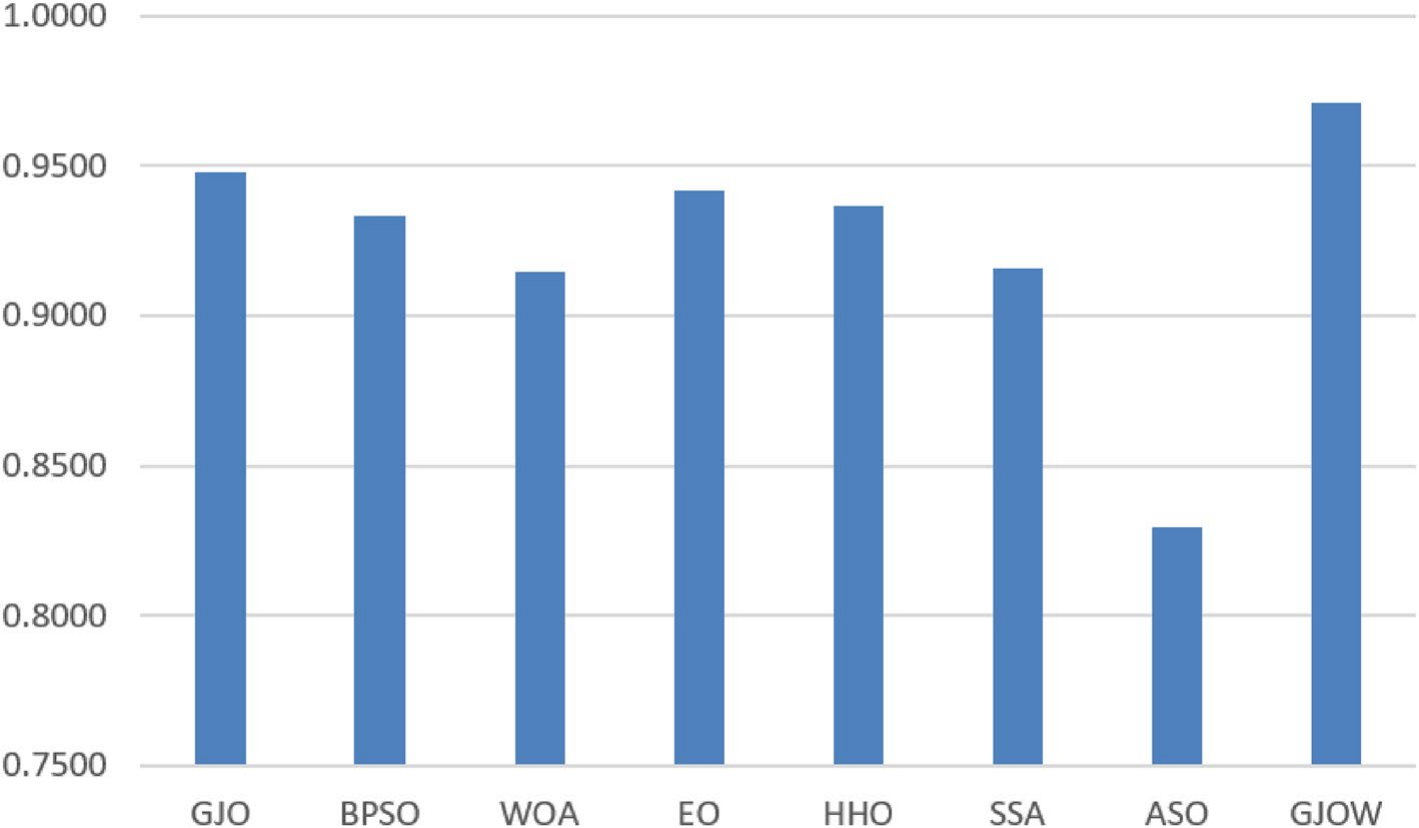
Average of accuracy measure for the datasets.

Furthermore, the effectiveness of the suggested method was assessed using three of the classification measures. In this regard, Table 5 shows the results of the accuracy measure. In Table 6, the suggested GJOW achieved the best classification accuracy results in all datasets. It achieved the best accuracy both when used alone in eleven datasets and when combined with other approaches in the remaining datasets. The GJO obtained second place followed by the EO method. The BPSO came in the third rank followed by HHO, SSA, and WOA, respectively. The ASO approach indicated the values with the lowest classification accuracy. Figure 6 illustrates the classification accuracy’s average. In this figure, the algorithms names are listed in x-axis, while the average values of the classification accuracy measure are listed in y-axis. The longer bar (i.e. GJOW) is for the better algorithm whereas, the shorter bar indicates the worst algorithm.

Table 7 illustrates the sensitivity measure’s results. Of the Nineteen datasets used for this measurement, the suggested GJOW had the best sensitivity, and it came in the first place. The GJO also came in second place followed by the EO method. The BPSO and HHO obtained the third and fourth ranks respectively. The lowest sensitivity values were also shown by the ASO method.

The GJOW also displayed the finest outcomes in the specificity measure as in Table 8, it got the best specificity in twenty datasets and was ranked first whereas, both EO and HHO came in the second and third ranks respectively followed by the GJO, BPSO, SSA, and WOA.

These results show that the suggested GJOW can correctly categorize various dataset types and achieve the highest classification accuracy when compared to other methods.

In addition, Table 9 presents the statistical results of each feature selection (FS) method using the Friedman test. According to the table, it is evident that the proposed GJOW method attained the highest rank compared to all other methods, exhibiting a significant difference. It attained the first position in terms of accuracy, sensitivity, and specificity measures, while EO and GJO followed closely in second and third place, respectively.

### Parameter analysis of GJOW

In this section, we investigate the performance of GJOW using different values for its parameters: c1 and b. The results of this experiment are presented in Tables 10 and 11. We consider values of c1 as 1.2, 1.5, and 1.7, and values of b as 0.5, 1, and 2.

**Table 10:** The results of different values of the parameter c1.

c1	Accuracy			Sensitivity			Specificity		
	1.2	1.5	1.7	1.2	1.5	1.7	1.2	1.5	1.7
base_Brain_T21	**1.0000**	**1.0000**	**1.0000**	**1.0000**	**1.0000**	**1.0000**	**1.0000**	**1.0000**	**1.0000**
base_Brain_T91	0.9167	**1.0000**	0.9167	**1.0000**	**1.0000**	**1.0000**	**1.0000**	**1.0000**	**1.0000**
base_Breastcancer4	0.9786	0.9714	**0.9857**	**0.9890**	0.9878	0.9877	0.9592	0.9655	**1.0000**
base_BreastEW	0.9737	0.9825	**0.9912**	**1.0000**	**1.0000**	**1.0000**	**0.9804**	0.9767	0.9787
base_CongressEW1	0.9425	**0.9770**	**0.9770**	0.9636	0.9672	**1.0000**	0.9062	**1.0000**	**1.0000**
base_Exactly	**1.0000**	**1.0000**	**1.0000**	**1.0000**	**1.0000**	**1.0000**	**1.0000**	**1.0000**	**1.0000**
base_HeartEW2	**0.8889**	**0.8889**	0.8519	0.9259	**0.9655**	0.9000	**0.9259**	0.9200	0.7917
base_IonosphereEW5	0.9577	**0.9859**	0.9718	**1.0000**	**1.0000**	**1.0000**	**0.9394**	0.9200	0.9091
base_KrvskpEW4	**0.9781**	0.9703	0.9531	**0.9909**	0.9632	0.9626	0.9904	0.9936	**0.9655**
base_leuk1	**1.0000**	**1.0000**	**1.0000**	**1.0000**	**1.0000**	**1.0000**	**1.0000**	**1.0000**	**1.0000**
base_Lymphography3	0.9000	**0.9333**	**0.9333**	**1.0000**	**1.0000**	**1.0000**	**1.0000**	0.8333	**1.0000**
base_M-of-n3	**1.0000**	**1.0000**	**1.0000**	**1.0000**	**1.0000**	**1.0000**	**1.0000**	**1.0000**	**1.0000**
base_PenglungEW3	**1.0000**	**1.0000**	**1.0000**	**1.0000**	**1.0000**	**1.0000**	**1.0000**	0.9286	1.0000
base_SonarEW2	**1.0000**	**1.0000**	**1.0000**	**1.0000**	**1.0000**	**1.0000**	**1.0000**	**1.0000**	**1.0000**
base_SpectEW2	**0.8889**	**0.8889**	**0.8889**	**1.0000**	**1.0000**	0.9000	**1.0000**	**1.0000**	0.9545
base_Tic-tac-toe3	0.8281	**0.8385**	0.8333	0.8800	0.8833	**0.9154**	0.7612	**0.7639**	0.6935
base_Vote3	0.9667	0.8385	0.9833	**1.0000**	**1.0000**	**1.0000**	0.9355	0.9773	0.9756
base_WaveformEW9	0.7620	0.7680	**0.7690**	0.7589	0.7699	**0.7768**	0.8705	**0.8769**	0.8750
base_WineEW1	**1.0000**	**1.0000**	**1.0000**	**1.0000**	**1.0000**	**1.0000**	**1.0000**	**1.0000**	**1.0000**
Zoo	**1.0000**	**1.0000**	**1.0000**	**1.0000**	**1.0000**	**1.0000**	0.9167	**1.0000**	**1.0000**

Best values are in bold.

**Table 11:** The results of different values of parameter b.

	Accuracy			Sensitivity			Specificity		
b	0.5000	**1.0000**	2.0000	0.5000	1.0000	2.0000	0.5000	1.0000	2.0000
base_Brain_T21	**1.0000**	**1.0000**	**1.0000**	**1.0000**	0.8333	**1.0000**	**1.0000**	**1.0000**	**1.0000**
base_Brain_T91	0.9091	**1.0000**	**1.0000**	**1.0000**	**1.0000**	0.6667	**1.0000**	0.9000	0.6667
base_Breastcancer4	0.9714	0.9643	**0.9786**	**0.9897**	0.9574	0.9890	0.9767	**0.9783**	0.9388
base_BreastEW	**0.9825**	**0.9825**	**0.9825**	0.9868	**1.0000**	**1.0000**	**1.0000**	0.9762	0.9750
base_CongressEW1	**1.0000**	0.9655	**1.0000**	**1.0000**	0.9434	**1.0000**	**1.0000**	**1.0000**	**1.0000**
base_Exactly	**1.0000**	**1.0000**	**1.0000**	**1.0000**	**1.0000**	**1.0000**	**1.0000**	**1.0000**	**1.0000**
base_HeartEW2	0.8704	0.8333	**0.9259**	0.8966	0.9091	**0.9697**	0.9200	**0.9524**	0.9048
base_IonosphereEW5	**1.0000**	0.9859	0.9577	**1.0000**	**1.0000**	**1.0000**	**1.0000**	**1.0000**	0.9310
base_KrvskpEW4	0.9812	**0.9875**	0.9656	0.9769	**0.9815**	0.9784	0.9863	**1.0000**	0.9873
base_leuk1	**1.0000**	**1.0000**	**1.0000**	**1.0000**	**1.0000**	**1.0000**	**1.0000**	**1.0000**	**1.0000**
base_Lymphography3	0.9333	0.9655	**1.0000**	**1.0000**	**1.0000**	**1.0000**	0.9333	0.9375	**1.0000**
base_M-of-n3	**1.0000**	**1.0000**	**1.0000**	**1.0000**	**1.0000**	**1.0000**	**1.0000**	**1.0000**	**1.0000**
base_PenglungEW3	**1.0000**	**1.0000**	**1.0000**	**1.0000**	**1.0000**	**1.0000**	**1.0000**	0.9286	**1.0000**
base_SonarEW2	**1.0000**	**1.0000**	0.9762	**1.0000**	**1.0000**	**1.0000**	**1.0000**	**1.0000**	**1.0000**
base_SpectEW2	**0.9444**	0.8704	0.9074	**1.0000**	0.9333	0.8462	0.9773	**1.0000**	**1.0000**
base_Tic-tac-toe3	0.8542	0.8542	**0.8594**	0.9083	**0.9185**	0.9127	0.7917	**0.8596**	0.7576
base_Vote3	**1.0000**	0.9833	0.9667	**1.0000**	**1.0000**	**1.0000**	**1.0000**	0.9730	0.9737
base_WaveformEW9	0.7800	0.7610	**0.7910**	**0.7614**	0.7455	0.7543	0.8843	**0.8910**	0.8869
base_WineEW1	**1.0000**	**1.0000**	**1.0000**	**1.0000**	**1.0000**	**1.0000**	**1.0000**	**1.0000**	**1.0000**
zoo	**1.0000**	**1.0000**	**1.0000**	**1.0000**	**1.0000**	**1.0000**	0.6923	**1.0000**	**1.0000**

Best values are in bold.

**Figure 7:**
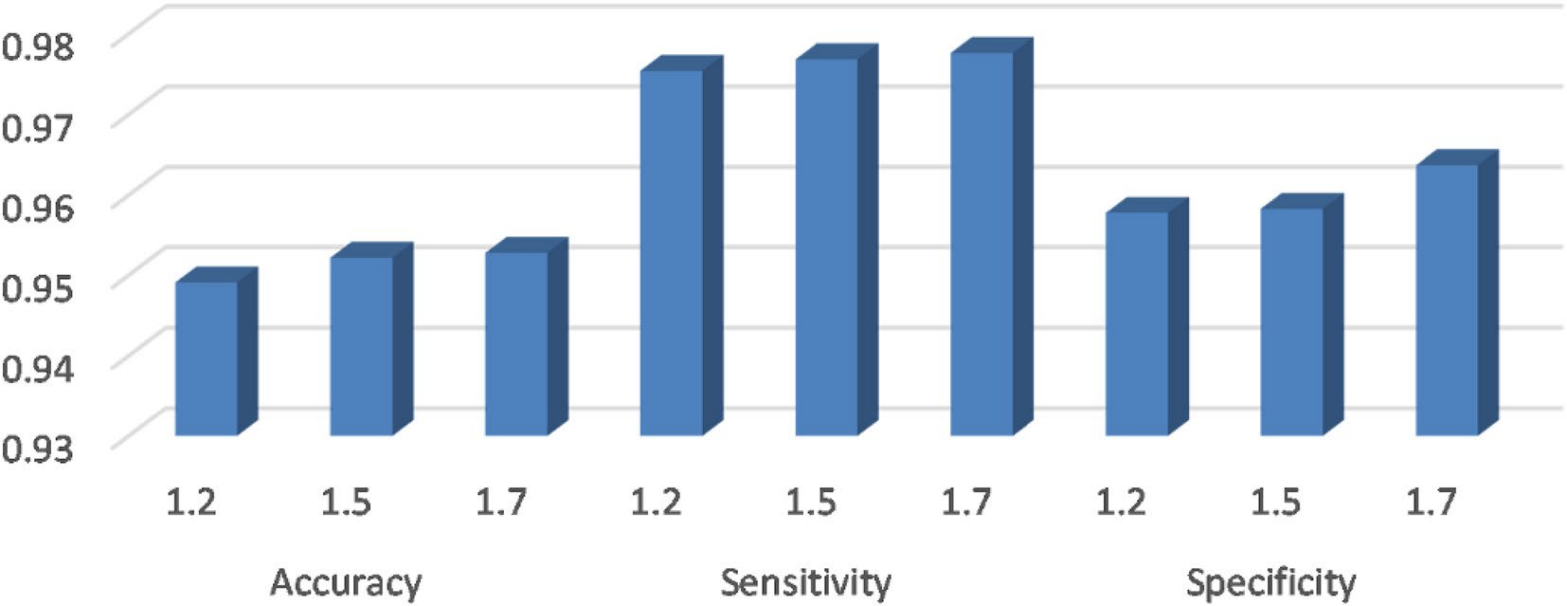
Average performance at different values of parameter c1.

Table 10 shows that the accuracy of GJOW with c1 values of 1.5 and 1.7 is nearly the same and achieves the best performance across all datasets. However, the accuracy remains consistent for all c1 values across approximately 10 datasets. In terms of Sensitivity and Specificity, GJOW performs best with c1 values of 1.7 and 1.2, respectively. The average performance across the tested datasets is depicted in Fig. 7, which indicates that increasing the value of c1 improves the overall performance. However, the difference in performance between c1 = 1.5 and c1 = 1.7 is negligible.

Furthermore, analyzing the results of GJOW with different values of parameter b (0.5, 1, and 2) as shown in Table 11, we observe the following points. Firstly, the best overall performance is achieved with a b value of 2, suggesting that increasing the value of b enhances the prediction ability of GJOW while keeping c1 fixed at 1.5. Overall, there is no significant difference in the results obtained by varying the parameters c1 or b, as the differences are minimal between them.

### Discussion of bone metastasis datasets

For further analysis, the suggested GJOW was evaluated using real medical data described in Sect. “Golden jackal optimization”. The outcome is listed in Table 12. The results of different measures for the objective function are shown in Table 12. This table demonstrates that the suggested GJOW indicated promising outcomes in average, min, max, and standard deviation measures. It came in the first rank with a small difference with HHO and WOA methods. The ASO displayed the worst results among all methods. Additionally, the suggested GJOW obtained the best accuracy and specificity measures.

**Table 12:** Performance of GJOW using bone metastasis datasets.

		GJO	BPSO	WOA	EO	HHO	SSA	ASO	GJOW
Fitness	MAX	0.3829	0.4171	0.3615	0.3731	0.3385	0.4274	0.9923	**0.3206**
	MIN	0.3678	0.4166	0.3077	0.3303	0.2847	0.4267	0.4177	**0.2344**
	STD	0.0046	**0.0002**	0.0200	0.0132	0.0164	0.0002	0.1746	0.0181
	AVG	0.3809	0.4168	0.3297	0.3677	0.3163	0.4272	0.6867	**0.2775**
Accuracy		0.6581	0.6496	0.6496	0.6239	0.6752	0.6239	0.6239	**0.7350**
Specificity		0.8767	0.8630	0.7808	**1.0000**	0.7534	**1.0000**	**1.0000**	**1.0000**

Best values are in bold.

From the results and discussion of the developed method we can be observed its high ability to detect Bone Metastasis Detection, as well as the high ability to classify the UCI datasets. However, the time complexity of the developed method is considered one of the main limitations that still suffers from it. In addition, the initial population has largest effect on the convergence rate of the GJOW towards the optimal features.

## Conclusion and future work

Early identification of bone cancer is crucial due to its hazardous nature. However, accurately detecting the disease poses a significant challenge. This paper addresses this challenge by investigating the effectiveness of an adaptive algorithm called GJOW in improving the accuracy of bone cancer detection in bone scans through feature extraction and selection. The proposed method aims to determine the presence or absence of a tumor in bone scans that have been classified as normal or abnormal. The study contributes to the field by introducing a machine learning-based approach for bone metastasis detection using gamma camera scans, enhancing the Golden Jackal Optimization (GJO) algorithm by incorporating operators from the Whale Optimization Algorithm (WOA), and evaluating a developed feature selection method using real-world bone metastasis datasets. The experimental results highlight the success of the GJOW algorithm, which achieves high classification accuracy. Notably, the proposed method outperforms others across all datasets, with an average accuracy of 97% in the first experiment and the best accuracy of 73% in the second experiment. Future work will involve leveraging a larger dataset to further evaluate the model's performance and exploring additional feature selection methods.

## Data Availability

The data used in the study are available from the corresponding authors upon request.

## References

[1] Coleman R (2001). Metastatic bone disease: Clinical features, pathophysiology and treatment strategies. Cancer Treat. Rev..

[2] Lukaszewski B (2017). Diagnostic methods for detection of bone metastases. Contemp. Oncol. (Pozn).

[3] Elfarra FG, Calin MA, Parasca SV (2019). Computer-aided detection of bone metastasis in bone scintigraphy images using parallelepiped classification method. Ann. Nucl. Med..

[4] Zheng Q (2021). Artificial intelligence performance in detecting tumor metastasis from medical radiology imaging: A systematic review and meta-analysis. EClinicalMedicine.

[5] Salman I (2018). Impact of metaheuristic iteration on artificial neural network structure in medical data. Processes.

[6] Sharma A (2021). Bone cancer detection using feature extraction based machine learning model. Comput. Math. Methods Med..

[7] Nasef MM, Eid FT, Sauber AM (2020). Skeletal scintigraphy image enhancement based neutrosophic sets and salp swarm algorithm. Artif. Intell. Med..

[8] Liu WC (2021). Machine learning for the prediction of bone metastasis in patients with newly diagnosed thyroid cancer. Cancer Med..

[9] Nakajima K (2013). Enhanced diagnostic accuracy for quantitative bone scan using an artificial neural network system: A Japanese multi-center database project. EJNMMI Res..

[10] Avula, M., Lakkakula, N.P. and Raja, M.P. Bone Cancer Detection from MRI Scan Imagery Using Mean Pixel Intensity. In *2014 8th Asia Modelling Symposium*, 141–146 (2014).

[11] Ranjitha, M. et al. Bone cancer detection using K-means segmentation and Knn classification. In *2019 1st International Conference on Advances in Information Technology (ICAIT)* (IEEE, 2019).

[12] Sinthia P, Sujatha K (2016). A novel approach to detect bone cancer using k-means clustering algorithm and edge detection method. Asian Res. Publ. Netw. ARPN J. Eng. Appl. Sci..

[13] Satheesh Kumar B, Sathiyaprasad B (2021). Bone Cancer Detection Using Feature Extraction with Classification Using K-Nearest Neighbor and Decision Tree Algorithm.

[14] Zhang Y (2019). A filter-based bare-bone particle swarm optimization algorithm for unsupervised feature selection. Appl. Intell..

[15] Jabber, B. et al. SVM model based computerized bone cancer detection. In *2020 4th International Conference on Electronics, Communication and Aerospace Technology (ICECA)* (IEEE, 2020).

[16] Shukla A, Patel DA (2020). Bone cancer detection from X-ray and MRI images through image segmentation techniques. Int. J. Recent Technol. Eng. (IJRTE).

[17] Mohammadzadeh H, Gharehchopogh FS (2020). A multi-agent system based for solving high-dimensional optimization problems: A case study on email spam detection. Int. J. Commun. Syst..

[18] Gharehchopogh FS (2023). Advances in sparrow search algorithm: A comprehensive survey. Arch. Comput. Methods Eng..

[19] Piri J (2022). Feature selection using artificial Gorilla troop optimization for biomedical data: A case analysis with COVID-19 data. Mathematics.

[20] Shishavan ST, Gharehchopogh FS (2022). An improved cuckoo search optimization algorithm with genetic algorithm for community detection in complex networks. Multimed. Tools Appl..

[21] Gharehchopogh FS (2023). Quantum-inspired metaheuristic algorithms: Comprehensive survey and classification. Artif. Intell. Rev..

[22] Shen Y (2023). An improved whale optimization algorithm based on multi-population evolution for global optimization and engineering design problems. Expert Syst. Appl..

[23] Ayar M (2021). Chaotic-based divide-and-conquer feature selection method and its application in cardiac arrhythmia classification. J. Supercomput..

[24] Gharehchopogh FS (2023). Slime mould algorithm: A comprehensive survey of its variants and applications. Arch. Comput. Methods Eng..

[25] Mohammed H, Rashid T (2023). FOX: A FOX-inspired optimization algorithm. Appl. Intell..

[26] Abdullah JM, Ahmed T (2019). Fitness dependent optimizer: Inspired by the bee swarming reproductive process. IEEE Access.

[27] Mohammadi M (2019). Donkey and smuggler optimization algorithm: A collaborative working approach to path finding. J. Comput. Des. Eng..

[28] Mohammed HM, Umar SU, Rashid TA (2019). A systematic and meta-analysis survey of whale optimization algorithm. Comput. Intell. Neurosci..

[29] Abdulhameed S, Rashid TA (2021). Child drawing development optimization algorithm based on child’s cognitive development. Arab. J. Sci. Eng..

[30] Rahman CM, Rashid TA (2021). A new evolutionary algorithm: Learner performance based behavior algorithm. Egypt. Inform. J..

[31] Hama Rashid DN, Rashid TA, Mirjalili S (2021). ANA: Ant nesting algorithm for optimizing real-world problems. Mathematics.

[32] Mirjalili S, Lewis A (2016). The whale optimization algorithm. Adv. Eng. Softw..

[33] Chopra N, Mohsin Ansari M (2022). Golden jackal optimization: A novel nature-inspired optimizer for engineering applications. Expert Syst. Appl..

[34] Liebgott A (2018). ImFEATbox: A toolbox for extraction and analysis of medical image features. Int. J. Comput. Assist. Radiol. Surg..

